# Relationship between hepatitis B DNA viral load in the liver and its histology in patients with chronic hepatitis B 

**Published:** 2015

**Authors:** Tahmineh Biazar, Yousef Yahyapour, Mohammad Reza Hasanjani Roushan, Ramazan Rajabnia, Mahmoud Sadeghi, Hasan Taheri, Mohammad Ranaei, Masomeh Bayani

**Affiliations:** 1 Infectious Diseases and Tropical Medicine Research Center, Babol University of Medical Sciences, Babol, Iran.; 2 Department of Pathology Babol University of Medical Sciences, Babol, Iran.

**Keywords:** Chronic hepatitis B, Liver, Viral load, Serum

## Abstract

**Background:**

Serial measurement of hepatitis B virus (HBV) DNA levels in the liver and its relation with liver damage and serum HBV DNA levels are guide to begin and/or end a treatment course. This study evaluated the relationship between liver hepatitis B DNA viral load with liver histology in patients with chronic hepatitis B (HBV).

**Methods:**

Thirty patients with chronic anti-Hbe positive hepatitis B, with liver enzymes ≥ 2 times of the upper limit of normal and positive HBV DNA of any amount were entered in the study. They underwent percutaneous liver biopsy. Liver and serum viral load were determined using real time polymerase chain reaction method (RT-PCR). Liver function tests and liver histology for all cases were recorded. The amount of viral load in the liver and histological grading and staging were recorded. Data were collected and analyzed.

**Results:**

The mean age of the patients was 32.8±10 years and 24 (80%) patients were males. Ten (33.3%) patients had HBV viral load levels less than 20000 IU/mL. There was a significant correlation between liver viral load levels with staging or grading of liver damage.

**Conclusion:**

The results of the present study showed a strong correlation between liver viral load and liver damage in patients with chronic hepatitis B.

Chronic hepatitis B (HBV) is a growing worldwide public health issue. Its prevalence and the mode of transmission of the virus varies greatly in different parts of the world (1, 2). Chronic infection hepatitis B with virus affects approximately 350 million people worldwide and is the most common cause of viral liver disease, cirrhosis and hepatocellular carcinoma, accounting for over 1 million deaths annually (3, 4). The natural course of chronic hepatitis B virus infection had four phases including: immune-tolerant phase immune-clearance, inactive carrier phase and reactivation (5). There is a high level of HBV replication, HbeAg positivity, and a normal or minimally elevated alanine transaminase (ALT) in the ‘immunotolerant phase’. During the ‘immune clearance phase’, there is a reduction in HBV DNA levels and increased liver inflammation. The occurrence of seroconversion from eAg positive to anti-e antibody is usually followed by a decrease in viral replication and ALT in inactive phase and finally, HBV replication <2000 IU/ml is associated in most patients with biochemical and histological regression of inflammatory activity (6).

High HBV DNA viral replication represents disease activity in both HBeAg positive and negative of chronic disease and it is a main independent risk factor in comparison to Hbe Ag, ALT and cirrhosis for hepatocellular carcinoma (HCC) (6, 7). Several studies have shown that HBV DNA levels may not always be associated with the destruction of liver tissue (8, 9). Therefore, measurement of serum HBV DNA in the liver and its correlation with liver damage and serum HBV DNA levels may guide clinicians for the beginning and ending of treatment. Appropriate antiviral therapy can reduce the incidence of viral resistance and failure of treatment. This study was conducted to evaluate the relationship between serum and liver hepatitis B virus DNA and liver histology in patients with chronic hepatitis B.

## Methods

This study was done on 30 patients with anti-HBe positive chronic hepatitis B who were referred to the Infectious Diseases Clinic of Babol University of Medical Sciences between 2010 and 2014. Inclusion criteria were patients with chronic hepatitis B, anti-HBe (+), have elevated liver enzymes and positive HBV DNA of any amount. Patients with chronic hepatitis C, alcoholic hepatitis, a history of recent drug hepatitis, autoimmune hepatitis and jaundice, ascites and those who had received any previous treatment for hepatitis B were excluded. Liver biopsy was performed for histological examination of the liver and the determination of HBV DNA levels.

**Histological examination**: After injecting 5-10cc Lidocaine 2% in the liver, percutaneous biopsy was performed using gauge needle number 16, the samples were put in formalin 10 % and sent to the department of pathology for pathological examination and also the other samples were sent to the laboratory for the measurement of HBV DNA viral loads.

**Deparaffinization of specimens**: Paraffinated blocks from all the liver samples were cut in 5 µm sections and 10 sections were collected in the same microcentrifuge tube. Samples were deparaffinated in xylene 3 times. Tissue dehydration was done with absolute ethanol according to our previous procedure.

**DNA Extraction**: After tissue digestion with proteinase K, DNA was isolated using High Pure PCR Template preparation kit (Roche Diagnosis GmbH, Mannheim, Germany) according to manufacturer's instructions.

**Quantitative Real-Time PCR**: was performed using the COBAS TaqMan HBV test (Roche Diagnostics, abaranchburg.N.J.) (cut off value, 35 copies/ml equivalent to 6 IU/mL), according to the manufacturer’s instructions. Instead of using 100 µl of serum for detection.100 µl of diluted DNA extract was utilized to the sample lysis buffer.

**Statistical Analysis: **Continuous variables (serum viral load, liver viral load, AST, ALT, grading and staging of hepatic involvement) were analyzed using Mann-Whitney and Kruskal-Wallis test and Kendall’s correlation coefficient where appropriate P-value of <0.05 was considered to be statistically significant.

## Results

The mean age of the patients was 32.2±10 years (ranged 18 to 57 years). Twenty-four (80%) patients were males and 6 patients (20%) were females. Ten (33.3%) of the subjects had a serum viral load of less than 20000 IU/mL and 20 (66.7%) had serum viral load greater than 20000 IU/ml. The mean of liver viral load was 769.8±1199.7 copies/ml. All non-hepatitis B patients had undetectable serum HBV DNA levels. The mean AST and ALT levels was 47±19.4 and 81±51.2 IU/l, respectively. The mean grading and staging was 4.6±2.1 and 1.7±2, respectively.

**Figure 1:**
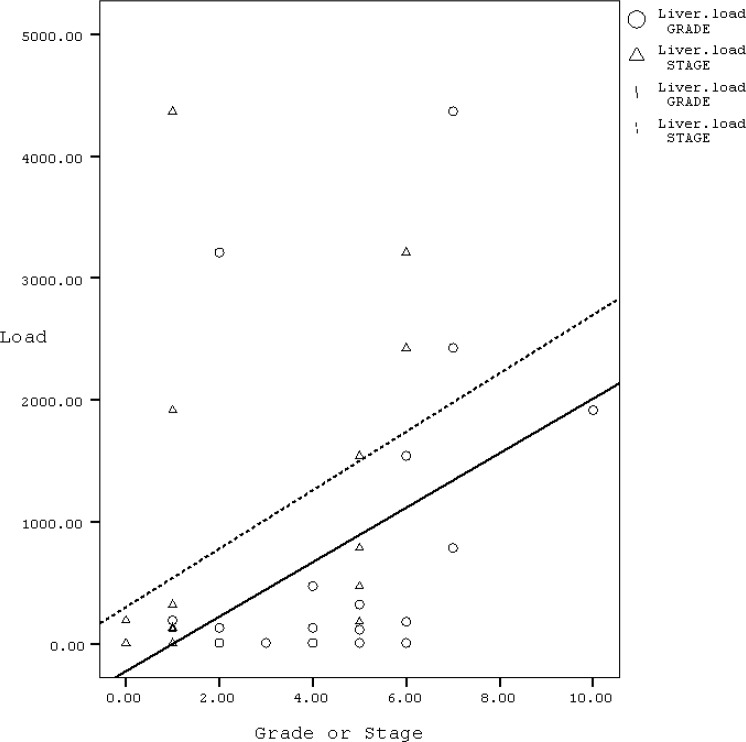
The correlation between HBV liver viral load with grade and stage of chronic hepatitis B

The mean viral loads of the liver in those with serum viral loads of less and more than 20000IU/ml was 186.6 and 941.41 copies/ml, respectively (P=0.12). There was no correlation between age or sex of these patients with their AST or ALT or liver histology and viral loads of liver and serum. The correlation between HBV liver viral loads of chronic hepatitis B patients with grading, staging and liver enzymes are shown in table 1 and figure 1. There was significant correlation between liver viral load with grading and staging of liver damage in patients with chronic hepatitis. 

All of the patients had minimal and mild necroinflammation and only one patient had moderate necroinflammation and none had severe necroinflammation or marked fibrosis.

However, there was not significant relation between liver viral load and AST, likewise between liver viral load and ALT.

**Table 1:** The correlation between HBV liver viral load of CHB patients with grading, staging and liver function tests

	**Kendal correlation coefficient**	**P-value**
AST	0.055	0.73
ALT	- 0.114	0.473
Grade	0.353	0.044
Stage	0.585	0.002

## Discussion

In spite of the accessibility of many commercial tests for the determination of HBV DNA levels in serum, there are no standardized methods in determining HBV DNA levels in liver biopsy samples in chronic hepatitis B patients (10). Therefore, the use of standard methods for the determination of HBV DNA levels in liver can be more important. This study confirmed a suitable method for the quantitation of HBV DNA in liver.

This study was performed to investigate the relationship between hepatitis B virus DNA in serum and liver in 30 patients with chronic hepatitis B. 

The results showed that 10 (33.3%) patients had serum viral load of less than 20000, and 20 cases had viral load more than 20000 IU/ml. 

In the present study, there was a significant correlation between liver viral load of chronic hepatitis B patients with grade and stage of liver damage. In a study performed by Alam et al. in 2011, 499 patients with chronic hepatitis B were studied. Among the HBeAg-negative patients, 66 (23.1%) patients had histological activity index (HAI) more than 4 and 31 (10.8%) had fibrosis (11). In another study by Xu et al. in 2008, 233 patients with chronic hepatitis B were studied. In HBeAg-positive patients, 46% had grades three and four and in HBeAg-negative patients, 52% were in grades three and four, and these differences were statistically significant (12). In another study by Wong et al. in 2004, intrahepatic viral load has also significant correlation with the degree of fibrosis that was similar to our study (13).

Results show that there was no significant correlation observed between serum viral load and liver viral load. In Lee et al.’s study in 2002, 33 patients with chronic hepatitis was observed and there was not a significant correlation between serum and liver viral load (14), which is similar to the findings of our study. In the study of Wong et al. in 2004, intrahepatic HBV DNA was positive when serum HBV DNA was negative which is similar to our findings (13). Wong’s study and our study results may support the old findings that in Asian patients, sometimes even with very low HBV DNA level, disease progression will continue (15-18).

In conclusion, the results of the present study showed a strong correlation between liver viral load with liver damage in patients with chronic hepatitis. It seems that there was a difference between the real viral load in the blood and liver. Also, due to no significant relationship between serum and liver viral load can be discussed that in HbeAb or precore mutant patients, measuring only serum viral load may not be helpful, investigation should be focused on histology and liver function test of patients.

## Notes

**Funding: **This study is a thesis project of Tahmineh Biazar (No: 303).
